# Protective and Restorative Effects of Nutrients and Phytochemicals

**DOI:** 10.2174/1874091X01812010046

**Published:** 2018-04-17

**Authors:** Tania Rescigno, Mario F. Tecce, Anna Capasso

**Affiliations:** Department of Pharmacy, University of Salerno, Fisciano (SA), Italy

**Keywords:** Antioxidants, Bioactive nutrients, Health, Phytochemicals, Angiogenesis, Carcinogens activation

## Abstract

**Intoroduction:**

Dietary intake fundamentally provides reintegration of energy and essential nutrients to human organisms. However, its qualitative and quantitative composition strongly affects individual’s health, possibly being either a preventive or a risk factor. It was shown that nutritional status resulting from long-term exposition to specific diet formulations can outstandingly reduce incidences of most common and most important diseases of the developed world, such as cardiovascular and neoplastic diseases. Diet formulations result from different food combinations which bring specific nutrient molecules. Numerous molecules, mostly but not exclusively from vegetal foods, have been characterized among nutritional components as being particularly responsible for diet capabilities to exert risk reduction. These “bioactive nutrients” are able to produce effects which go beyond basic reintegration tasks, i.e. energetic and/or structural, but are specifically pharmacologically active within pathophysiological pathways related to many diseases, being able to selectively affect processes such as cell proliferation, apoptosis, inflammation, differentiation, angiogenesis, DNA repair and carcinogens activation.

**Conclusion:**

The present review was aimed to know the molecular mechanisms and pathways of activity of bioactive molecules; which will firstly allow search for optimal food composition and intake, and then use them as possible therapeutical targets and/or diagnostics. Also, the present review discussed the therapeutic effect of both nutrients and phytochemicals.

## 1 INTRODUCTION

Accumulating evidence suggests that a regular consumption of dietary botanicals, including cruciferous vegetables such as cabbage and broccoli, *Allium* vegetables such as garlic and onion, green tea, *Citrus* fruits, soybeans, tomatoes, berries, and ginger, as well as medicinal plants and substances from marine environment is associated with reduced risks of developing chronic diseases such as cardiovascular diseases and cancer [1, 2]. This association has been partly ascribed to the presence of a variety of phytonutrients naturally occurring in plant-based foods [3-5]. Several of these bioactive compounds, such as curcumin (from the rhizome of Curcuma longa), genistein (from soybeans), lycopene (from tomatoes), isothiocyanates such as phenethyl isothiocyanates (PEITC), benzyl isothiocyanates (BITC), sulforaphane (SFN), dithiolethiones (from cruciferous vegetables), and resveratrol (from grapes and peanuts) show strong anticarcinogenic properties both in preclinical or clinical trials [6]. Each phytochemical molecule most likely interacts with more than one molecular target, thereby influencing different signalling pathways and the expression of a large variety of genes [3-5]. These include blockage of metabolic activation and/or DNA binding of carcinogens, stimulation of detoxification, repair of DNA damage, suppression of cell proliferation and angiogenesis or metastasis, induction of differentiation or apoptosis of precancerous or malignant cells, etc [3-5, 7]. Phytochemicals that influence multiple signaling pathways often enhance the activity of conventional chemotherapy and radiation therapy, and may be used in lower doses when such synergistic effects are present [8, 9]. Importantly, oxidative stress and inflammatory tissue injuries are two of the most critical factors that are involved in multistage carcinogenesis. One of the most important links between inflammation and cancer is proinflammatory transcription factor NF-kB. NF-kB is a ubiquitous and evolutionarily conserved transcription factor that regulates the expression of genes involved in the transformation, survival, proliferation, invasion, angiogenesis and metastasis of tumor cells. Accordingly, many research laboratories have shown that nutraceuticals (*e.g*., curcumin, sulforsaphane, genistein, resveratrol, epigallocathechin gallate, etc.) can exert anticancer activity by suppressing the NF-kB signaling pathway.

## 2 CURCUMIN

Curcumin, (diferuloylmethane), derived from the ancient Indian medicine turmeric, is a widely studied nutraceutical. This perennial herb has multiple ingredients, including curcuminoids, the most active ingredient for medicinal use [10]. Curcumin has been used for a variety of diseases, including respiratory diseases, inflammation, liver and hepatic disorders, obesity, diabetic wounds, rheumatism and certain malignant tumors [11-15]. In addition, no studies in animals and humans have demonstrated significant toxicity related to curcumin, even at very high doses [16, 17]. Recently, it was shown that curcumin exerts preventive as well as therapeutic effects in different experimental models of carcinogenesis. For example, it was reported that curcumin inhibits expression of cyclooxygenase-2 (COX-2) in mouse skin treated with the tumor promoter 12-O-tetradecanoylphorbol-13-acetate (TPA) via inactivation of the eukaryotic transcription factor NF-kB. Interestingly, inhibition of NF-kB by curcumin appears to be mediated by blocking ERK1/2 and p38 MAPK [18]. In addition, when human colonic epithelial cells were pretreated with curcumin, inhibition of tumor necrosis factor (TNF)-α-induced cyclooxygenase 2 (COX-2) gene transcription and NF-kB activation was observed [19]. Curcumin suppressed IkB degradation by down-regulation of the NF-kB-inducing kinase (NIK) and IkB kinase (IKK). Curcumin has also been reported to inhibit IkBα phosphorylation in human multiple myeloma cells [20] and murine melanoma cells [21] through suppression of IKK activity, which contributed to its antiproliferative, proapoptotic and antimetastatic activities. This compound also acts as a chemopreventive agent because of its *in vivo* regression of various animal models of colorectal carcinogenesis. Accordingly, oral administration of curcumin inhibits azoxymethane (AOM)-initiated and dextran sulphate sodium (DSS)-promoted colorectal carcinogenesis in mice [22]. In this animal model, cyclic administration of DSS in drinking water results in the establishment of chronic colitis and the development of colorectal dysplasia and cancers with pathological features that resemble those of human colitis-associated neoplasia [22]. Villegas *et al* [23] have recently demonstrated the protective effect of dietary curcumin in this model of chronic colitis-associated colorectal carcinoma (CRC) through a reduction in β-catenin expression and in the production of the proinflammatory cytokines TNF-α and IFN-γ. Since adenomas are generally considered as precursor lesions of colorectal cancer, various reports have examined the effect of dietary curcumin in the adenomatous polyposis coli (Apc^Min+^) mouse, a model of human familial adenomatous polyposis. These animals are genetically predisposed to develop intestinal tumors as a result of a mutation of the Apc gene [24]. The findings of these studies have shown that curcumin retarded adenoma growth, reflected by a total number of adenomas and mean adenoma size [25, 26]. This effect may be mediated by the inhibition of COX-2 protein expression and the reduction of levels of two oxidative stress DNA adducts in intestinal adenoma tissue [27]. Curcumin also decreased expression of the oncoprotein β-catenin in the enterocytes of the Apc^Min+^ mouse [25]. This bioactive compound also acts as a potent inducer of apoptosis in cancer cells. Curcumin induces upregulation of proapoptotic proteins such as Bax, Bcl-2-interacting mediator of cell death (Bim), Bak, p53 upregulated modulator of apoptosis (Puma), and PhoRbol-12-myristate-13-acetate-induced protein 1 (Noxa) and downregulation of the antiapoptotic proteins Bcl-2 and Bcl-xL [28, 29]. In gastric cancer cells, curcumin was also shown to suppress the transition of cells from the G1 to S phase, which was accompanied by a decrease in cyclin-D1 and p21-activated kinase activity [30]. In Hep2 human laryngeal cancer cells, curcumin also exerted an inhibitory effect on the tumor cell invasion and metastasis that were associated with downregulated MMP-2 expression and reduced activity and expression of integrin receptors, FAK and membrane-type 1 MMP [31]. A role of curcumin in the regulation of tumor cell angiogenesis has also been revealed. Specifically, curcumin was found to completely prevent induction of VEGF synthesis in microvascular ECs stimulated with glycation end products, which was mediated by downregulation of NF-kB and AP-1 activity [32]. Curcumin also inhibited angiogenesis through the mediation of angiopoietins 1 and 2, HIF-1, and heme oxygenase 1 (HO-1) in cancer cells [33]. Finally, *in vitro* studies with curcumin have also demonstrated that this bioactive compound acts synergistically with 5-fluorouracil plus oxaliplatin, a standard chemotherapy for this malignancy, in inhibiting the growth of HCT-116 and HT-29 cells through attenuation of surface growth factor pathways, specifically EGFRs and insulin-like growth factor-1 receptor (IGF-1R), which are considered to play an important role in the progression of CRC [34]. Similarly, curcumin can be also used in combination with other chemotherapeutic agents and radiation to minimize toxicity in head and neck cancer [35]. Another interesting study suggests that cotreatment of curcumin and resveratrol was more effective than either agent alone in inhibiting the growth of p53-positive (wt) and p53-negative colon cancer HCT-116 cells *in vitro* and *in vivo* in SCID xenografts of colon cancer HCT-116 (wt) cells [36].

## 3 ISOFLAVONES

Asian diet contains high amounts of soy products, which are rich in isoflavones, plant-derived polyphenols. Soy intake has long been recognized to reduce the incidence of different malignant tumors, cardiovascular disease, postmenopausal syndrome, diabetes mellitus and osteoporosis [37-39]. **Soy isoflavones** are dietary agents structurally related to 17-β-estradiol and show some estrogenic or anti-estrogenic properties. They are referred to as phytoestrogens. Their consumption has been associated with a reduced risk of some hormone-dependent diseases [40]. One the most abundant and the best–characterized dietary isoflavones is **genistein** [39]. Like other isoflavones, genistein is capable of binding to the estrogen receptor, with a preference for receptor β, the predominantly expressed receptor subtype in the gastrointestinal tract and to trigger mechanisms of estrogen action [41]. Recently, Ariazi EA *et al*. [41], have also shown that genistein exerts beneficial anti-inflammatory effects in a rodent model of TNBS-induced chronic colitis through reduction in myeloperoxidase (MPO) activity and COX-2 mRNA protein expression. Likewise, quercetin, genistein and apigenein have also shown to induce apoptosis in various cancer cells [42, 43]. For example, several in vitro studies have demonstrated that genistein has anti-cancer effects in prostate, breast, colon gastric, lung and pancreatic adenocarcinomas and in lymphoma [44]. Consistent with this notion, Raffoul JJ *et al*. [45], have also reported that genistein significantly inhibited NF-kB activity in human prostate cancer PC3 cells leading to altered expression of cell cycle regulatory proteins, thus promoting cell cycle arrest. In addition, genistein also increased the expression of cleaved PARP leading to apoptosis in prostate cancer cells. Previous studies by Yu Z *et al*. [46], have also suggested that genistein-induced apoptosis through the overexpression of Bax and the down-regulation of Bcl-2 in human colon cancer HT-29. Another report by Ouyang G *et al*. [47], demonstrated that genistein present in soybeans, induced DNA damage and apoptosis in human ovarian cancer cells through phosphorylation and activation of p53 and a decrease in the ratio of Bcl-2/Bax, and phosphorylated AKT levels. A number of studies have also revealed a role of genistein in the regulation of tumor cell invasion. Specifically, genistein inhibited cell adhesion to vitronectin and cell migration of invasive breast cancer cells by inhibiting the transcriptional activity of AP-1 and NF-kB, resulting in the suppression of u-PA secretion from cancer cells [48]. Finally, genistein has also been shown to suppress metastasis in different cancer types, including colon cancer. Genistein reduced experimental lung metastasis of murine colon 6-L5 carcinoma cells by 44% [49]. It has been suggested that epigenetic modifications, such as DNA promoter methylation and histone modification, play crucial roles in regulating the expression of many metastasis suppressor genes, which suggests the association between aberrant epigenetic alterations and cancer metastasis [50].

## 4 RESVERATROL

Another promising nutraceutical capable of targeting various steps in tumor cell development is resveratrol (3,5,4’- trihydroxy-trans-stilbene). This bioactive compound, which belongs to the group of stilbenes, is a natural polyphenolic phytoalexin originally isolated from white hellebore that is present in grape skins, red wine, cranberries, mulberries and peanuts [51]. It is well documented that resveratrol exerts potent antioxidant, anti-aging, anti-inflammatory and anti-cancer effects, promotes vascular endothelial function and enhances lipid metabolism [52-54]. The highly potent inhibitory effects of resveratrol against tumorigenesis suggest that it is an efficient chemopreventive agent for cancer. For example, resveratrol ameliorated DSS-induced acute inflammation in mouse colonic mucosa through inhibition of iNOS expression and NF-kB, signal transducer and activator of transcription-3 (STAT3) and extracellular signal-regulated kinase (ERK) activity [55] demonstrated that oral administration of resveratrol ameliorated established chronic colitis in IL-10 (-/-) mice by inducing immunosuppressive CD11b(+) Gr-1(+) myeloid-derived suppressor cells (MDSCs) in the colon.. These authors also reported that resveratrol decreased local and systemic inflammatory cytokine concentrations, including IL-1 β, IL-6, TNF-α, IL-12, IFN-γ, and RANTES [56]. Other studies also reported that resveratrol suppressed colon cancer cell proliferation and elevated apoptosis after IGF-1 exposure through suppression of IGF-1R/Akt/Wnt signaling pathways as well as activation of p53 [57]. Moreover, this polyphenol significantly decreased the amount and proportion of β-catenin in the nucleus of colon cancer cell line RKO [58]. Zykova TA *et al*. [59], also reported that the anticancer effects of resveratrol were mediated directly through COX-2 and that this bioactive compound decreased COX-2 mediated PGE_2_ production in HT-29 cells. Additionally, resveratrol has also been shown to exert antimetastatic effects via its strong inhibition of cell adhesion, migration and invasion, as well as reduction of secretion of MMP-9 and MMP-2 in Lovo cells cultured under normoxia and hypoxia [60]. Kimura Y *et al*. [61], also suggested that antimetastatic effects of resveratrol were probably due to the inhibition of VEGF-induced angiogenesis. Early work published by Yu R *et al*. [62] clearly suggested that resveratrol attenuated phorbol ester and UV induced AP-1 activity in human cervical cancer (HeLa) cells and did so by interfering with the MAPK cascade. In animals studies, Schneider Y *et al*. [63], demonstrated that orally administrated resveratrol decreased the number of tumors in the small intestine and prevented tumor formation in the colon of Apc^Min+^ mice. Moreover, these authors showed that resveratrol downregulated genes known to be implicated in cell cycle progression, such as cyclins D1 and D2, as well as upregulated a panel of genes controlling the activation of immune response and the inhibition of the carcinogenic process and tumor expansion. Clinical studies by Nguyen *et al*. [64], reported that resveratrol in combination with other bioactive compounds in grape powder (GP) inhibited Wnt pathway in the normal colonic mucosa, as indicated by a reduction in the expression of a panel of Wnt target genes. Another recent report by Patel KR *et al*. [65], showed that in patients with confirmed colorectal cancer, who were to undergo surgical resection of their malignancy, consumption of eight daily doses of resveratrol at 0.5 or 1.0 g before surgery, significantly reduced colon tumor cell proliferation, as reflected by reduction in Ki-67 staining, a surrogate marker of cell growth. A role of resveratrol in the regulation of tumor cell angiogenesis has also been found. Resveratrol is able to suppress the growth of new blood vessels in animals. It directly inhibits capillary endothelial cell growth and blocks both VEGF and FGF-receptor-mediated angiogenic responses through inhibition of phosphorylation of MAPK in ECs [66]. Studies also suggest resveratrol reduced the migratory and invasive abilities of A549 lung cancer cells and was associated with inhibition of NF-kB activation and expression of MMP-2 and MMP-9 [67]. Interestingly, resveratrol has biphasic effects over low to high spectrum of concentrations. Studies have shown that at a lower dose, resveratrol acts as an anti-apoptotic agent, providing cardioprotection as evidenced by increased expression in cell survival proteins, improved postischemic ventricular recovery and reduction of myocardial infarct size and cardiomyocyte apoptosis and maintains a stable redox environment compared to control [68]. Contrary, at a higher dose, resveratrol acts as a pro-apoptotic compound, inducing apoptosis in normal and cancer cells by exerting a death signal [69]. At higher doses, resveratrol, also depresses cardiac function, elevates levels of apoptotic protein expressions, results in an unstable redox environment, increases myocardial infarct size and number of apoptotic cells [70]. One study from France shows that at higher dose (60μM), resveratrol inhibits the growth and induces apoptosis in case of both normal (60 μM) and leukemic (5-43 μM) hematopoietic cells [71]. Additionally, resveratrol also induced apoptosis in human multidrug-resistant SPC-A-1/CDDP cells associated with a downregulation in survivin [72]. Similarly, resveratrol was shown to induce apoptosis and suppress constitutive NF-kB in rat and human pancreatic carcinoma cell lines [73]. Mammary tumors isolated from rats treated with resveratrol displayed reduced expression of COX-2 and MMP-9 accompanied by reduced NF-kB activation [74]. Treatment of human breast cancer MCF-7 cells with resveratrol also suppressed NF-kB activation and cell proliferation [74]. Collectively, these findings suggest that, at a lower dose, resveratrol can be very useful in maintaining the human health whereas, at a higher dose, this bioactive compound exerts pro-apoptotic actions on healthy cells, but can kill tumor cells.

## 5 CAROTENOIDS

Carotenoids are another class of the natural chemopreventive agents which have received increasing attention because of the decreased incidence of cancers associated with their consumption [75]. These pigments are lipid components present in variable quantities in fruits and vegetables as well as algae and non-photosynthetic organisms such as animals, fungi and bacteria. Among the carotenoids, there are six found predominantly in human plasma: β-carotene, α-carotene, lycopene, lutein/zeaxanthin, astaxanthin, and β-cryptoxanthin. The first three belong to the carotene sub-group, while the last three are xanthophylls [76]. Due to their extended conjugation systems, carotenoids and xanthophylls (oxocarotenoids) are very efficient scavengers of singlet oxygen and, under low oxygen tension, peroxyl radicals [77]. However, under certain experimental conditions, they can also act as pro-oxidants [78]. Intervention studies in humans with carotenoid-rich diets have shown that these compounds exert photoprotection of the skin as measured by decreased sensitivity to UV radiation-induced erythema [77-83]. Importantly, in these studies, dietary intake of tomato paste (40 g/day, equivalent to 16 mg lycopene/day) over a period of 10 weeks led to a 40% reduction in skin erythema development induced by exposure to solar-simulating UV radiation [82]. Similar, erythema development was diminished in subjects whose diets were supplemented with β-carotene (24mg/day) or a carotenoid mixture consisting of β-carotene, lutein and lycopene (8 mg each/day) for 12 weeks [83]. Interestingly, in these studies protection correlated with an increase in the carotenoid levels in skin and serum. It should also be pointed out that the protective properties of carotenoids are probably due to their potent antioxidant activity and their ability to induce cellular protective responses through the Nrf2/ARE pathway. Thus, dietary carotenoids and their metabolites induce phase 2 cytoprotective enzymes [84] and share with all other classes of phase 2 inducers a common chemical property, the ability to react with sulfhydryl groups [84]. Previous *in vitro* studies also showed that β-carotene exhibited growth-inhibitory and proapoptotic effects in human colon adenocarcinoma cells, at least in part, by a reduction in the expression of COX-2 [85]. In addition, data from animal models of colon carcinogenesis have shown that dietary supplementation of β-carotene reduced the number of ACF induced by AOM and colonic COX-2 expression [86]. Lycopene, a dietary constituent present in tomatoes, red fruits and vegetables, was recently reported to inhibit migration and invasion of hepatoma cell line SK-Hep-1, which was associated with upregulation of a metastasis suppressor gene nm23-H1 [87]. Lycopene and β-carotene both have also been shown to inhibit metastasis in an experimental setting. The inhibition of lung metastasis by β-carotene has been evaluated with B16F-10 melanoma cells in C57BL/6 mice. In this study, after tumor induction, administration of β-carotene decreased the formation of tumor nodule, collagen hydroxyproline in the metastasized lung, lung hexosamine content, uronic acid, serum sialic acid and gamma-glutamyl transpeptidase. These endpoints correlated with the improved histopathology of lung tissue with the administration of β-carotene [88]. The inhibition of lung metastasis by β-carotene has been also shown with human hepatoma SK-Hep1-1 cells. In this study, human hepatoma SK-Hep1-1 cells were injected into athymic nude mice through the tail vein, and it was found that lycopene reduced the tumor number and cross-sectional area in the lung. This bioactive compound also reduces the level of vascular endothelial growth factor (VEGF) and metalloproteinase [89]. Additionally, these results also suggest that β-carotene has a higher efficacy than lycopene in the inhibition of lung metastasis, taking into consideration the net increase of the two phytochemicals in the lungs and the factors associated with tumor invasion, proliferation and angiogenesis [89, 90].

## 6 FATTY ACIDS

Fatty Acids are considered another important source of biological components for the treatment of several pathologies, much of them being inflammatory diseases, including inflammatory bowel disease (IBD), atherosclerosis, Parkinson’s and Alzheimer’s diseases and cancer. Many of these compounds are long chain fatty acids where they can be either saturated or unsaturated, with polyunsaturated fatty acids (PUFAs) being the most studied for their pharmacological potential [91, 92]. Fish oils are the main sources of N-3 PUFAs because of their high levels of docosahexaenoic acid (DHA), eicosapentaenoic acid (EPA) and docosapentaenoic acid (DPA). Data from epidemiological, clinical and experimental studies have suggested that dietary fish oil containing omega-3 fatty acids exerts benefits for human health with relevant preventive effects on cardiovascular diseases [91, 92], also including protection against colon cancer development and other cancers such as endometrial, breast and prostate cancer [93-96]. The marine environment and especially microalgae, are another huge source of different fatty acids, including monounsaturated fatty acids, such as oleic acid (OLA) and PUFAs, including linoleic acid (LNA), α-linolenic acid (ALA), eicosapentaenoic acid (EPA) and docosahexaenoic acid (DHA). All of these fatty acids have been shown to be involved in inflammatory signaling pathways and many studies reported their antioxidant, anti-inflammatory and anti-cancer activities, both *in vitro* and *in vivo* [96, 97]. Interestingly, many fatty acids are known to bind to PPAR-γ, with PUFAs having higher binding affinity than saturated or monounsaturated fatty acids [98]. Current studies on PUFAs from microalgae suggest that these compounds, called oxylipins (oxidative product of fatty acids), exert an interesting anti-inflammatory activity *in vitro* by decreasing TNF-α production. This anti-inflammatory effect may be due, in part to the regulation of PPAR-γ and NF-kB pathway [99]. A number of studies suggest that N-3 PUFAs exert beneficial biological functions in carcinogenic processes. *In vitro* studies have reported that EPA and DHA induced apoptosis in the colon cancer cell lines HT-29, Caco-2, and DLD-1 through an increase in caspase-3 activity and inhibition of Bcl-2 [100]. Another study reported that lycopene and EPA synergistically inhibited the proliferation of human colon cancer HT-29 cells by downregulation of PI3K/Akt/mTOR signaling pathway [101]. Allred CD *et al*. [102], have also demonstrated that growth inhibitory effects of EPA in these cells were the result of a PPARγ-mediated pathway. Other authors [103] suggested that DHA reduced colon tumor growth *in vitro* through p53 dependent –apoptosis and p53 independent pathways. In addition, they reported an inhibition of the growth of human adenocarcinoma COLO 205 in nude mice by a diet supplemented by golden algae oil containing DHA [103]. Current studies by Cao W *et al*. [104], also showed that PUFAs shift estrogen signaling to suppress human breast cancer cell growth. Specifically, these authors, suggest that EPA and DHA shifted the prosurvival and proliferative effect of estrogen to a pro-apoptotic effect in human breast cancer (BCa) MCF-7 and T47D cells. 17 β-estradiol (E2) enhanced the inhibitory effect of N-3 PUFAs on BCa cell growth. In contrast, in cells treated with stearic acid (SA) as well as cells not treated with fatty acid, E2 promoted breast cancer cell growth. Subsequent studies by the same authors demonstrated that G protein-coupled estrogen receptor 1(GPER1) might mediate the pro-apoptotic effect of estrogen. N-3 PUFA treatment initiated the pro-apoptotic signaling of estrogen by increasing GPER1-cAMP-PKA signaling response and blunting EGFR, Erk 1/2, and AKT activity. Collectively, these results not only provide the evidence to link N-3 PUFAs biologic effects and the pro-apoptotic signaling of estrogen in breast cancer cells, but also shed new insight into the potential application of N-3 PUFAs in BCa treatment. Preclinic studies by Mohammed A *et al*. [105], also suggest that diets rich in N-3 PUFAs may be beneficial for prevention of pancreatic cancer. The health benefits of N-3 PUFAs are thought to stem mainly from the EPA and DHA metabolites, type 3 series eicosanoids (*e.g*., PGE_3_) compared to arachidonic acid (AA) metabolites of pro-inflammatory type 2 series eicosanoids (*e.g*., PGE_2_) [106, 107]. Experiments were designed using N-3 fatty acid desaturase (Fat-1) transgenic mice, which can convert N-6 PUFAs to N-3 PUFAs endogenously, to determine the impact of N-3 PUFAs on pancreatic intraepithelial neoplasms (PanINs) and their progression to pancreatic ductal adenocarcinoma (PDAC). This genetic approach of modifying FA composition by converting N-6 to N-3 FAs endogenously not only effectively increases the absolute amount of N-3 FAs but also significantly decreases the level of N-6 FAs, leading to a balanced ratio of N-6 to N-3 FAs in the pancreas. In addition, this allows production of two different FA profiles (high versus low N-6/N-3 ratios) in the animals without or with the Fat-1 gene by using just a single diet, thus eliminating the potential diet variations. Importantly, significant reductions of pancreatic ducts with carcinoma and PanIN 3 lesions were observed in the compound transgenic mice. The levels of N-3 PUFAs were much higher in the pancreas of compound transgenic mice than in those of mice harboring a conditional K-ras mutant allele (LSL-Kras^G12D/+^) in combination with a pancreas-specific Cre recombinase transgene (p48^Cre/+^). In addition, the molecular analysis of the pancreas showed a significant down-regulation of proliferating cell nuclear antigen (PCNA)COX-2), cyclooxygenase-2 (COX-2), 5-lipoxygenase (5-LOX), 5-LOX-activating protein, Bcl-2, and cyclin D1 expression levels in Fat-1-p48^Cre/+^ -LSL- Kras^G12D/+^ mice compared to p48^Cre/+^ -LSL- Kras^G12D/+^ mice. Collectively, these results suggest that Fat-1 gene expression inhibits pancreatic tumor cells proliferation, induces tumor cell apoptosis, and alters AA metabolism. In addition, these data also indicate that endogenous N-3 PUFAs delay the progression of PanIN-1 and PanIN-2 to PanIN-3 and PDAC. Therefore, elevating N-3 PUFAs may be an important strategy to delay/prevent pancreatic cancer in high-risk patients. The main characteristic of cancer cachexia is the progressive loss of muscle mass [108], leading to sarcopenia, which in turn translates into clinically relevant negative consequences. Inflammatory cytokines play an important role in the pathogenesis of cancer cachexia [109]. As a consequence, the anti-inflammatory effects of omega-3 fatty acids may be of benefit in the prevention and treatment of cancer cachexia [110].

## 7 GREEN TEA

Green tea (*Camellia sinensis*) is one of the most widely consumed beverages in the world and epidemiologic studies have suggested varying effects on cancer incidence [111, 112]. Among the green tea constituents, epigallocatechin gallate (EGCG) is the major biologically active compound, which has been shown to possess *in vitro* and *in vivo* anti-inflammatory and anti-oxidant properties, responsible for their cancer chemopreventive potency [113, 114]. Recently, Abboud PA *et al*. [115], demonstrated the beneficial effects of EGCG in different experimental models of colitis. These effects were associated with a significant reduction of NF-kB and (AP-1) activation, as well as an inhibition of TNF-α. and IFN-γ production. EGCG also exerted antioxidant activity by reducing nitric oxide (NO) and malondialdehyde (MDA) and increasing superoxide dismutase (SOD) in colonic mucosa [116]. In addition, epigallocatechin gallate ameliorated acute experimental colitis by the suppression of macrophage and mast cells activities [117]. Navarro-Peràn E *et al*. [118], have also demonstrated that the anti-inflammatory properties of EGCG may be related to its antifolate activity. In fact, EGCG, by blocking folic acid uptake, can disturb the metabolism of this vitamin in Caco-2 cells, inducing the release of adenosine and the suppression of NF-kB activation. Protein kinases, which phosphorylate a specific substrate, play crucial roles in the regulation of multiple cell signaling pathways and cellular functions. However, deregulation of protein kinases under certain pathological conditions leads to perturbation of protein kinase-mediated cell signaling pathways and results in various disorders including inflammation and cancer [119]. He Z *et al*. [120], showed that tea flavonoid epigallocatechin gallate (EGCG) also has direct binding properties to kinases. This binding of Fyn kinase by EGCG could inhibit the phosphorylation of EGF-induced p38, activating transcription factor-2 (ATF-2) and STAT1 with attenuated cell transformation. *In vitro* studies have also reported that EGCG induces apoptosis in multiple cancer cells [121-123]. For example, EGCG has been shown to induce apoptosis by suppression of COX-2 expression and the subsequent production of PGE_2_ in different colon cancer cells, such as SW837, HT-29 and HCA-7 cells [121-123]. The inhibitory effect of EGCG on the expression of this enzyme was mediated by the reduction of NF-kB activity [124] and activation of AMP-activated protein kinase (AMPK), a positive regulator of autophagy [123, 125]. The stimulation of AMPK was accompanied by the reduction of VEGF and glucose transporter, Glut-1, in EGCG-treated cancer cells [123]. In another study, EGCG has also been reported to promote apoptosis in T24 human bladder cancer cells by modulating PI3K/Akt signaling pathway and Bcl-2 family proteins [126]. IGF-1R has emerged as a key therapeutic target in many malignancies, including childhood cancers such as Ewing family tumors (EFT). EGCG was found to inhibit survival of EFT through inhibition of IGF-1R activity, induction of apoptosis via up-regulation of Bax, and decreased expression of Bcl-2, Bcl-XL, and myeloid cell leukemia (Mcl)-1 proteins [127]. Additionally, EGCG also plays an important role in the regulation of tumor cell angiogenesis. Consistent with this notion, EGCG inhibited production of VEGF and IL-8 from normal human keratinocytes [128-130]. In human colon cancer cells, EGCG attenuated VEGF production through inhibition of ERK-1 and ERK-2 kinases [131-133]. Recently, Tang FY *et al*. [134], have also reported that EGCG inhibited ephrinA1-mediated endothelial cell (EC) migration as well as tumor angiogenesis through inhibition in ERK-1/ERK-2 activation. Finally, it has been also shown that EGCG blocked HGF-induced invasion and metastasis of hypopharyngeal carcinoma cells. In these cells, HGF was shown to promote the autophosphorylation of c-Met and HGF receptor, activate Akt and Erk pathway, and enhance the activity of matrix metalloproteinase (MMP)-9 and urokinase-type plasminogen activator. These combined effects lead to cancer cell proliferation, migration and invasion of tumors. It is noteworthy that EGCG at a physiologically relevant concentration (1μM) suppressed the molecular tumor motility and the molecular changes induced by HGF described. These results collectively indicate that EGCG may serve as a therapeutic agent to inhibit HGF-induced invasion in hypopharyngeal carcinoma patients [135].

## 8 CRUCIFEROUS VEGETABLES

Cruciferous vegetables such as broccoli, watercress, Brussels sprouts, cabbage, Japanese radish and cauliflower have been widely accepted as potential diet components that may reduce the risk of cancer [136]. Many of the chemopreventive effects of cruciferous vegetables are attributed to the isothiocyanates (ITCs) rather than their parent moiety, the glucosinolates. These compounds are not bioactive and appear to have no chemopreventive effects unless they are converted to ITCs and indole-3 carbinols by hydrolysis catalyzed by myrosinase. Disruption of plant cells during harvesting, processing, or chewing releases myrosinase which comes into contact with glucosinolates and hydrolyzes them to different ITCs [137]. Some isothiocyanates derived from cruciferous vegetables, such as phenethyl isothiocyanates (PEITC), benzyl isothiocyanates (BITC), and sulforaphane (SFN) have been found to be very potent chemopreventive agents in numerous animal carcinogenesis models as well as cell culture models. PEITC and SFN are multitargeted chemopreventive agents which exert their effects through the activation or inhibition of several cellular signaling pathways. The mechanisms of cancer chemopreventive activity of isothiocyanates are the inhibition of phase I enzymes cytochrome P-450s involved in the activation of carcinogen and/or induction of phase II detoxifying enzymes, such as quinone reductase, UDP-glucuronosyltransferase (UDP-GTs) and glutathione S-transferase (GSTs) through Nrf2-dependent pathway [138, 139]. For example, the isothiocyanate sulforaphane has been extensively investigated with regards to its ability to induce phase II detoxification enzymes [140]. It activates the Keap1/Nrf2/ARE pathway by chemically modifying highly reactive cysteine residues of Keap1, the cellular sensor for phase II inducers [141, 142]. As a consequence, Keap1 loses its ability to target transcription factor Nrf2 for ubiquitination and proteasomal degradation, resulting in its stabilization and nuclear translocation, where, in heterodimeric combination with a small Maf protein, it binds to the antioxidant response element (ARE) and activates transcription of phase II cytoprotective genes. In addition to being a potent activator of the Keap1/Nrf2/ARE pathway, SFN also inhibits proinflammatory responses [(i.e., lipopolysaccharide and interferon-γ- mediated elevation of inducible nitric oxide synthase (iNOS) and cyclooxygenase (COX-2)] at concentrations similar to those at which it induces cytoprotective responses [143]. Intraperitoneal injections of sulforaphane also stimulate both humoral and cell-mediated immunity in mice [144]. Sahu RP *et al*. [145], have also reported that ITCs exert antitumor activity, inhibiting the growth of various types of cultured human cancer cells. ITCs induce cell cycle arrest, cancer cell apoptosis [146], generation of reactive oxygen species (ROS) [145, 147], regulate the activation of transcription factors STAT3, NF-kB and Nrf2 [148-150], inhibit MAPK and PKC activities [145, 151], down-regulate estrogen receptor [152].

## 9 DITHIOLETHIONES

The dithiolethiones are another class of chemopreventive compounds isolated from cruciferous vegetables such as Brussels sprouts and cabbage. 3H-1,2-dithiole-3-thione (D3T) is a representative compound from these groups and has been studied extensively as cancer chemopreventive agent. In addition, synthetic substituted dithiolethiones such as oltipraz (5-[2-pyrazinyl]-4-methyl-1,2-dithiol-3-thione), and anethole dithiole (ADT) have been developed for pharmaceutical applications because of their antioxidant, radioprotective, chemotherapeutic and chemopreventive properties [153]. Although dithiolethiones have many mechanisms of action that may contribute to *in vivo* anticancer efficacy, they appear to act through alternate mechanisms including the inhibition of cell replication and induction of expression or activity of phase II enzymes such as GSTs [154]. Nrf2/Keap1/ARE signaling pathway is the most important signaling system in the induction of cytoprotective enzymes by chemopreventive dithiolethiones [155]. Consistent with this notion, dithiolethiones elevated transcript levels, protein levels and activities of multiple phase II genes in wild-type mice, but not in the homozygous Nrf2-mutant mice [156]. In addition, in animal models of carcinogenesis, dithiolethiones exert chemopreventive effects against the development of lung and other cancers [157]. Accumulating evidence suggests that the NF-kB signaling pathway regulates genes involved in tumor progression, angiogenesis and metastasis. Therefore, the NF-kB pathway has become an important target of cancer therapeutic/chemopreventive approaches. Recently, Switzer CH *et al*. [158], reported that the dithiolethiones ACS-1 and ACS-2 inhibited NF-κB transcriptional activity in estrogen receptor-negative breast cancer cells and murine tumor xenografts. Interestingly, this inhibition was not due to H(2)S release or protein phosphatase 2A activation, which are key properties of dithiolethiones, but occurred via a covalent reaction with the NF-κB p50 and p65 subunits to inhibit DNA binding. Dithiolethione-mediated inhibition of NF-κB-regulated genes resulted in the inhibition of interleukin (IL)-6, IL-8, urokinase-type plasminogen activator, and VEGF production. ACS-1 also inhibited matrix metalloproteinase-9 activity, cellular migration, and invasion, and ACS-2 reduced tumor burden and resulted in increased tumor-host interactions [158]. These results collectively suggest that dithiolethiones show potential clinical use for estrogen-negative breast cancer as a chemotherapeutic or adjuvant therapy.

## 10 GARLIC AND OTHER ALLIUM VEGETABLES

Convincing experimental evidence also suggests that garlic and other allium vegetables (*e.g*., onions) exert protective effects against a number of chronic diseases including cardiovascular problems, diabetes, infections and cancer [159-162]. Health benefits of garlic and other allium vegetables are mainly attributed to organosulphur compounds (OSCs), which are generated upon processing (cutting or chewing) of these edible plants [163]. These bioactive compounds include allicin, diallyl sulphide (DAS), diallyl disulfide (DADS) diallyl trisulfide (DATS), diallyl tetrasulfide as well as S-allylcysteine (SAC) or S-allylmercaptocysteine (SAMC), which are water-soluble compounds [164, 165]. Among the allium vegetable constituents, DATS is the major biologically active compound which has anticancer efficacy. Mechanisms underlying cancer chemopreventive effects of DATS revealed that this natural product targets multiple pathways to inhibit cancer development. These include potentiation of carcinogen detoxification [166-168], cell cycle arrest [169-173], induction of apoptosis [174, 175], suppression of oncogenic signaling [176], and inhibition of angiogenesis [177-179]. Milner and colleagues were the first to demonstrate anti-proliferative effects of DATS against cancer cells [174]. DATS-mediated suppression of cancer cell proliferation is associated with cell cycle arrest [170], which has been reported in gastric cancer cells [171], human liver cancer cells [170], colon cancer cells [172], prostate cancer cells [173]. Importantly, DATS-mediated G2/M phase cell cycle arrest in prostate cancer cells was associated with reactive oxygen species (ROS)–dependent hyperphosphorylation and destruction of the cell division cycle 25C (Cdc25C) phosphatase [173]. Notably, DATS-mediated G_2_ /M phase cell cycle arrest occurred selectively in cancerous cells because a normal prostate epithelial cell line (PrEC) was resistant to cell cycle arrest by DATS [73]. A few studies have looked at DATS-induced apoptosis *in vivo*. Importantly, DATS-induced apoptosis *in vivo* in PC-3 tumor xenografts correlated with a statistically significant increase in protein levels of Bax and Bak in the tumor [178]. Unlike cellular data [175, 180], however, the levels of Bcl-2, Bcl-XL or BID were not altered by DATS administration in PC-3 xenografts *in vivo* [178]. Additionally, DATS treatment also inhibited capillary-like tube formation and migration of human umbilical vain endothelial cells . Anti-angiogenic effects of DATS correlated with suppression of VEGF secretion, down-regulation of VEGF receptor-2 protein level and inactivation of Akt [177]. Mo SJ *et al*. [181], also reported that allicin, another important constituent of garlic, inhibited TNF-α-induced ICAM-1 expression in human umbilical endothelial cells (ECs) [181]. Similar studies also suggested that diallyl sulphide (DAS) reduced the serum level of VEGF in C57BL/6 mice bearing B16-F10 melanoma cells [182]. S-Allylcysteine and S-allylmercaptocysteine, obtained from garlic, instead suppressed the invasion ability of androgen-independent invasive prostate cancer cells [183] through restoration of E-cadherin expression. These results collectively suggest that OSCs, particularly DATS, can represent potential ideal agents in anticancer therapy, either alone or in association with other antitumor drugs [164-184].

## CONCLUSIONS AND REMARKS

The results of the present review indicate that nutrients and phytochemicals have a prominent role in the control of disease etiology and they may contribute as well to the improvement of human health through the regulation of specific processes. Thus, nutrients and phytochemicals may be considered as effective dietary signals, able to modify both metabolic programming and cell homeostasis by reducing disease risk with specific molecular mechanisms. The experimental and epidemiological studies described here, have emphasized the potential of nutrients and phytochemicals in the prevention or treatment of neoplastic and other several other diseases.

Given the above evidences, the nutrients and phytochemicals patterns may be considered as endogenous cellular mediators, since they influence gene, protein expression and metabolism as direct ligand or co-factor. Once its absorption at the cellular level, a nutrient or phytochemical can interact with specific signaling pathways and even a small change in its structure can differentially activate metabolic steps; fatty acids class and their level of carbon chain unsaturation can provide a proper example. The n-3 polyunsaturated fatty acids, in fact, promote anti-inflammatory pathways, whereas n-6 polyunsaturated fatty acids induce synthesis of pro-inflammatory molecules. Furthermore, *trans* fatty acids increase plasma levels of LDL-cholesterol, in contrast, n-3 polyunsaturated fatty acids do not.

The protective and restorative effects of nutrients and phytochemicals are likely to result from the modulation of distinct signal transduction pathways. Particularly, scientific literature confirmed that nutrients and phytochemicals are able to affect gene expression as consequence of a direct interaction with transcription factors by acting as a source of epigenetic modifications and by regulating the placement of these modifications [185].

Bioactive nutrients as resveratrol, curcumin, sulforaphane and tea polyphenols can modulate epigenetic patterns by altering the levels of S-adenosylmethionine and S-adenosylhomocysteine or directing the enzymes that catalyse DNA methylation and histone modifications. Aging and age-related diseases are associated with profound changes in epigenetic patterns, though it is not yet known whether these changes are programmatic or stochastic in nature [185].

Future work in this field seeks to characterize the epigenetic pattern of healthy aging to ultimately identify nutritional measures to achieve this pattern.
